# SENP Proteases as Potential Targets for Cancer Therapy

**DOI:** 10.3390/cancers13092059

**Published:** 2021-04-24

**Authors:** Paulina Tokarz, Katarzyna Woźniak

**Affiliations:** Department of Molecular Genetics, Faculty of Biology and Environmental Protection, University of Lodz, Pomorska 141/143, 90-236 Lodz, Poland; katarzyna.wozniak@biol.uni.lodz.pl

**Keywords:** SENP proteases, SUMOylation, deSUMOylation, DNA repair, cell cycle, cancer progression, SENP inhibitors

## Abstract

**Simple Summary:**

Post-translational modification—the biochemical addition of functional groups or proteins—occurs following protein biosynthesis and contributes to an increase in the functional diversity of the proteome. Post-translational modifications include SUMOylation—the covalent attachment of small ubiquitin-related modifier (SUMO) proteins to substrate proteins. SUMOylation is a reversible modification, which is erased by SUMO-specific proteases (SENPs). Deregulation of SENPs leads to cellular dysfunction and is associated with various diseases, including cancer. The role of SENPs in cancer pathogenesis is expected, and thus these proteins are considered promising targets for drug design and development. In this review, we will discuss the role of SENPs, focusing on DNA repair and the cell cycle—cellular pathways malfunctioning in most cancer cells—and provide an update on advances in the development of SENP-oriented inhibitors.

**Abstract:**

SUMOylation is a reversible post-translational modification (PTM) involving a covalent attachment of small ubiquitin-related modifier (SUMO) proteins to substrate proteins. SUMO-specific proteases (SENPs) are cysteine proteases with isopeptidase activity facilitating the de-conjugation of SUMO proteins and thus participating in maintaining the balance between the pools of SUMOylated and unSUMOylated proteins and in SUMO recycling. Several studies have reported that SENPs’ aberrant expression is associated with the development and progression of cancer. In this review, we will discuss the role of SENPs in the pathogenesis of cancer, focusing on DNA repair and the cell cycle—cellular pathways malfunctioning in most cancer cells. The plausible role of SENPs in carcinogenesis resulted in the design and development of their inhibitors, including synthetic protein-based, peptide-based, and small molecular weight inhibitors, as well as naturally occurring compounds. Computational methods including virtual screening have been implemented to identify a number of lead structures in recent years. Some inhibitors suppressed the proliferation of prostate cancer cells in vitro and in vivo, confirming that SENPs are suitable targets for anti-cancer treatment. Further advances in the development of SENP-oriented inhibitors are anticipated toward SENP isoform-specific molecules with therapeutic potential.

## 1. Introduction

**Figure 1:**
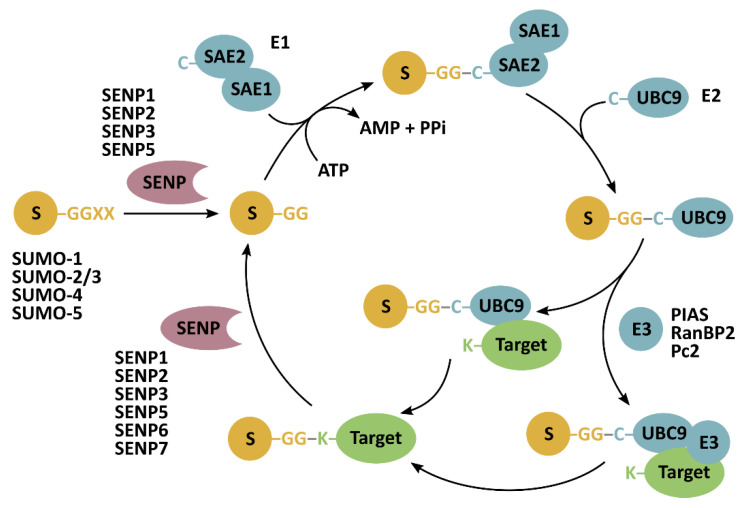
SUMOylation and deSUMOylation mechanism. Sentrin-specific proteases (SENP) have endopeptidase activity to cleave SUMO (S) preproteins, exposing their carboxy-terminal diglycine (GG) motif essential for their conjugation to lysine residues (K) in target proteins. SUMOylation is catalyzed by SAE1-SAE2 (heterodimeric SUMO-activating enzyme) (E1) and ubiquitin-conjugating enzyme 9 (UBC9) (E2). An E3-ligase can facilitate the last step of SUMO conjugation. SENPs isopeptidase activity allows for the release of SUMO from target proteins.

SUMO-mediated signaling is an entire proteome regulating pathway that has a crucial role in maintaining cellular physiology. SUMOylation is a highly dynamic, reversible modification, catalyzed by SUMO-specific activating (E1), conjugating (E2), and ligating (E3) enzymes (Figure 1). Deregulation of SUMOylation and deSUMOylation balance causes severe defects in cell proliferation and genome stability [1,2,3,4]. Therefore, it is not surprising that SUMOylation needs to be tightly regulated to prevent malignant transformation. SENPs conduct both processing of SUMO preproteins and deconjugation of SUMO from target proteins (Figure 1). The overexpression and genetic variation of SENPs have been reported in malignant cancers (Table 1). RNA-seq data from The Cancer Genome Atlas (TCGA) indicate that all SENPs are overexpressed in thyroid, lung, colorectal, head and neck, stomach, liver, pancreatic, renal, urothelial, prostate, testis, breast, cervical, endometrial, and ovarian cancers; glioma, and melanoma. The *SENP2* gene maps to chromosome 3q26-29, a region commonly amplified in epithelial cancers, including lung, esophagus, head and neck, cervical, and ovarian cancers [5]. Predominantly, the overexpression of SENPs is reported in cancer, but the down-regulation is observed in some types. TCGA also indicates that each SENP can serve as a prognostic marker for specific cancer types (Table 1). The diversity of cancers with dysregulated SENPs suggests that these proteases play a role in pathways generally malfunctioning in cancer rather than participating in tissue-specific pathways. Indeed, SENPs regulated such pathways as DNA repair, cell cycle, and neovascularization, indicating that the aberrant function of SENPs works as a cancer driver affecting the homeostasis of SUMO-mediated signaling. The study in mice demonstrated that prolonged SENP1 overexpression is critical for transforming the normal prostate gland, and gradually facilitates the onset of high-grade prostatic intraepithelial neoplasia [1]. Moreover, the overexpression of SENP1 was reported in precancerous prostate intraepithelial neoplasia in humans, confirming that SENP1 plays a role in cancer transformation. SENPs’ overexpression positively correlated with clinicopathological features such as TNM stage, tumor differentiation, lymph node metastasis, cancer aggressiveness, and recurrence (Table 1). In several cancers, SENPs overexpression can serve as a prognostic marker (Table 1). Mechanistic studies demonstrated that silencing SENPs suppresses cancer progression and metastasis [6,7,8,9,10,11,12,13,14]. The expression of SENPs can be endogenously regulated as demonstrated by the direct interaction between the 3′ untranslated region of SENP1 mRNA and microRNA-145 (miR-145), a significant miRNA tumor suppressor [4]. miR-145-mediated down-regulation of SENP1 induced quiescence of prostate cancer cells and reversed SENP1-promoted tumorigenesis in mice, pointing to miR-145 as a molecule of therapeutic value against cancer. Apart from slowing down tumor growth, silencing of SENP can sensitize cells to anticancer therapy. Knockdown of SENP6 induced radiosensitization of liver cancer cells [15].

## 2. SENP Proteases as Regulators of DNA Repair

SENPs are predominantly found in the nucleus and can thus regulate DNA damage response (DDR), including DNA repair and the cell cycle [23]. The DNA repair protein list has been well defined; however, little is known about how protein factors are regulated in a timely and accurate manner to ensure efficient DNA repair. SUMOylation is essential for the recruitment, activity, and clearance of several DNA repair factors [24]. Relatively little has been known about the role of enzymes that reverse SUMO conjugation in DNA repair. Multiple enzymes counter SUMO modifications, including enzymes belonging to the SENP family or SUMO-targeted ubiquitin ligases, to add further complexity to their interactions. The observations that the depletion of SENP causes specific DNA repair defects indicate that SENPs are critical regulators of DNA repair [2,5].

DNA double-strand breaks (DSBs) are one of the most deleterious lesions, which, unrepaired, could evoke genome instability and cancer. The participation of SENPs in the regulation of DSB repair has been reported. One of the first steps of DNA repair is chromatin relaxation, which is particularly important in heterochromatin to accumulate downstream repair factors. The long isoform of SENP7, SENP7L, localizes in a nucleus, particularly in heterochromatin, where it is associated with heterochromatin protein 1 alpha (HP1α) [25,26]. SENP7L participated in chromatin relaxation followed by homologous recombination (HR) through restricting the degree of polySUMOylation of local proteins, including KRAB-associated protein 1 (KAP1) [27]. SENPs promoted early DNA damage signaling and regulated DSB repair adaptor proteins’ recruitment, such as mediator of damage checkpoint 1 (MDC1) and TP53-binding protein 1 (53BP1). MDC1 interacted with γH2AX and coordinated DNA damage foci formation by retaining ATM to further propagate the DSB signal. While MDC1 recruitment is phosphorylation-dependent, its turnover at DSBs in G1 requires SUMOylation and ubiquitination. SENP2 protected MDC1 from excessive SUMOylation, which maintained MDC1 in DSB for non-homologous end joining (NHEJ) [5]. In the absence of SENP2, the hyperSUMOylated MDC1 was cleared from DSB. Similarly to MDC1, 53BP1 is one of the proteins initially recruited to DSB sites. It is a crucial regulator of DSBs’ repair pathway choice, which promoted NHEJ while competing with BRCA1 and inhibiting CtIP-mediated end resection in HR repair [28]. Efficient accumulation of 53BP1 at DSB sites required its SUMOylation [24]. SENP1 restored 53BP1 SUMOylation and promoted efficient NHEJ [29]. Although it seems counterintuitive that SENP1 allowed p53 SUMOylation, SENP1 could increase the free SUMO pool by the deSUMOylation of nucleoplasmic targets, thus allowing SUMO to conjugate to 53BP1. The SENP1-mediated regulation of 53BP1 indicated that SENPs not only ensured the efficient repair of breaks, but had also a role in switching between DNA repair sub-pathways.

SENPs regulate DNA repair executive proteins such as exonuclease I (EXO1) or replication protein A (RPA). EXO1 participates in extensive resection of DNA ends, which are essential intermediates for the downstream steps of HR repair. EXO1 constitutively interacted with SENP6, promoting its stabilization and the processing of DNA damage [30]. DSB end resection was ceased when EXO1 became SUMOylated and targeted for degradation. Apart from HR repair, EXO1 is implicated in other DNA repair pathways, including mismatch repair and the processing of stalled replication forks, indicating that SENPs could regulate a broader spectrum of DNA damage repair pathways. Replication protein A (RPA) is the main ssDNA binding protein complex, and it interacts with RAD51, HR recombinase. RPA70, the major ssDNA-binding subunit, was associated with SENP6, which maintained RPA70 in a hypoSUMOylated state [2]. In response to replication stress, SENP6 dissociated from RPA70, allowing its SUMOylation, which facilitated the recruitment of RAD51. Loss of SENP6 provoked an HR repair defect due to failure in RPA70 deSUMOylation and RAD51 filament formation.

Apart from DSB signaling conducted by the ATM-Chk2 pathway, another branch of DNA lesion signaling is ATR-Chk1. The proteomic profiling demonstrated that the ATR-Chk1 pathway was reported to be strongly influenced by SENP6 [31]. ATR-Chk1 is activated in response to DNA lesions that generate ssDNA. SENP6 was required for proper ATR-Chk1 activation and was a part of the hPSO4 complex, restricting its SUMOylation. ATR activation relies on the formation of the ATR-ATRIP heterodimer that binds to nucleofilaments composed of RPA and ssDNA. SENP6 deficiency impaired the chromatin association of the ATR cofactor ATRIP, thereby compromising the activation of Chk1. The chromatin association of ATRIP was controlled on the one hand by SENP6, and on the other by RNF4 (poly-SUMO-specific E3 ubiquitin ligase), indicating that SUMOylation of hPSO4 (which recruits ATRIP to DNA lesions) or other factors play a role in the proper activation of DNA damage signaling. Unexpectedly, the lack of SENP6 (which resulted in compromised ATR-Chk1 signaling) induced replication stress and DSBs, followed by Chk2 activation. Moreover, the interactome analysis revealed that other SENP6-associated proteins belong to ATM-Chk2-mediated HR repair, such as ATM, BRCA2, and DNA2. Altogether, these data indicate the prominent role of SENP6 in HR repair regulation.

Besides DSBs, DNA interstrand crosslinks (ICLs) are among the most detrimental DNA lesions, representing a significant challenge for DNA replication and transcription by preventing DNA strand separation. They are mainly repaired in either S phase, when the DNA replication forks stall at the ICLs, or at the actively transcribed regions in non-dividing cells. The repair of ICLs is conducted by the Fanconi anemia (FA) pathway, which encompasses 22 proteins from the FA family and other FA-like proteins. To repair ICLs, the FA pathway exploits other DNA repair pathways such as the nucleotide excision pathway (NER) and HR repair. The bioinformatical analysis revealed that SENP6 influenced the FA network by targeting FANCI and SLX4 and interacting with FANCI, FANCD2, FANCA, WDR48, FANCD1/BRCA2 [31]. Upon replication fork stalling, FA subunits FANCI and FANCD2 are SUMOylated in an ATR-dependent manner [32]. SUMO chain formation on FANCI/FANCD2 was limited by SENP6, which resulted in their lowered RNF4-mediated polyubiquitination and their maintenance on chromatin. SENP6 physically interacted with FA complex and deconjugated SUMO from FANCI [31]. Moreover, the lack of SENP6 resulted in reduced FANCD2 levels, suggesting that the SUMO-targeted ubiquitin ligases (STUbLs) pathway could degrade FANCD2. SLX4 (also known as FANCP) was highly SUMOylated in the absence of SENP6. SLX4 has been defined as a polySUMO-binding protein and a potential E3 SUMO ligase, and its SUMO-coupled function is implicated in response to replication stress [33]. Altogether, these data indicate that SENP6 regulated the function of multiple FA components.

All the above studies point out that SENPs have direct roles in DNA repair, including regulating chromatin relaxation, DNA damage signaling, assembly of DNA damage foci, recruitment of DNA repair factors, DNA repair factors chromatin assembly, and switching between DNA repair pathways and supplying and redistributing SUMO.

## 3. The Role of SENP Proteases in the Cell Cycle

SUMOylation and SENP-mediated deSUMOylation are highly dynamic throughout the cell cycle [3]. The depletion of SENPs followed by mitotic delay demonstrated their critical role in mitosis and meiosis [34,35,36]. Knockdown of SENP1 delayed sister chromatid separation at metaphase [34]. SENP1 translocated from the nuclear envelope and nuclear pore complex and accumulated at centrosomes, spindle microtubules, and kinetochores in mitosis. Similarly, SENP2 localized at kinetochores and its overexpression induced prometaphase arrest. Knockdown of SENP5 inhibited cell proliferation and resulted in binucleate cells formation, indicating a critical role of SENP5 in mitosis and/or cytokinesis [35]. Depletion of SENP6 evoked two distinct phenotypes, either permanent interphase arrest or mitotic delay [36]. The interphase arrest was associated with intact p53, suggesting the induction of the DNA damage response. The mitotic delay was associated with defects in spindle assembly and chromosome congression. Chromosome misalignment and missegregation were observed in SENP6 depleted cells. The mitotic delay was caused by the lack of SENP6-mediated protection of inner kinetochore proteins from degradation during the S phase, which was further reflected in the improper kinetochore assembly during mitosis. Altogether, these studies indicate a crucial role of SENPs in mitotic progression. The molecular mechanism of SENP-provided regulation of mitosis remains to be determined.

SENPs also play a role in cell cycle regulation through cyclin-dependent kinases (Cdks) [3]. Knockdown of SENP6 resulted in the activation of G1/S and G2/M checkpoints [2]. Cells expressing SENP3 mutant lacking phosphorylation sites demonstrated increased chromosome instability and were prone to tumorigenesis [37]. Cdk1 phosphorylated SENP3, which reduced SENP3 deSUMOylation activity at G2/M phase under normal conditions. In response to DNA damage, p53 suppressed the Cdk1-mediated phosphorylation of SENP3 [38]. The induction of SENP3 deSUMOylation activity was essential for the deconjugation of SUMO from Cdh1. Cdh1 deSUMOylation promoted its de-phosphorylation and cell cycle arrest at the G2/M checkpoint through Polo-like kinase 1 (Plk1)-Chk1 pathway.

## 4. The Role of SENPs in Cancer Progression

Tumor progression is associated with invasion, angiogenesis, and metastasis, and SENPs were reported to regulate these processes. Silencing SENP1 levels perturbed prostate and pancreas cancer cells’ ability to metastasize through the down-regulation of matrix metalloproteinase 9 (MMP9). Additionally to MMP9, SENP1 regulated the expression of MMP2 through the HIF-1α signaling pathway in prostate cancer [11]. Both MMP2 and MMP9 are highly expressed in cancer cells, and they participate in cancer invasion, angiogenesis, and metastasis. The interplay between SENP1 and HIF1α was explored in other studies, demonstrating a positive feedback loop between these proteins in liver and ovarian cancer [7,39]. SENP1 transcription in response to hypoxia was induced through hypoxia response element (HRE) in *SENP1* promoter in mice [14]. SENP1 was essential for stabilizing hypoxia-induced HIF-1α by deconjugating SUMO and preventing ubiquitin-mediated degradation in mice [40]. SENP1-mediated increased stabilization and transcriptional activity of HIF-1α led to enhanced liver cancer cell stemness and increased VEGF production and angiogenesis in endothelial cells [7,14]. These studies show a robust mutual interaction between SENP1 and HIF-1α and its role in cancer initiation, promotion, and progression. Apart from SENP1, HIF-1α transcriptional activity is regulated by SENP3, which serves as a redox sensor under oxidative stress through the deSUMOylation of p300—a HIF-1α coactivator in cervical cancer cells [41,42]. The overexpression of SENP3 induced VEGF mRNA expression and promoted angiogenesis in tumor xenografts. SENP1 deficiency increased SUMOylation of VEGFR2 and impaired its signaling, reducing pathological angiogenesis in endothelial cells [43]. Altogether, these data point to the role of SENPs’ regulation of cancer invasion, angiogenesis, and metastasis.

## 5. SENP Proteases Inhibitors

The aberrant expression of SENPs has been implicated in the pathogenesis of various cancers (Table 1). Mainly, numerous studies have been focused on the role of SENP1 in prostate cancer development. The Yeh group demonstrated that overexpression of SENP1 was present in more than 60% of samples of prostate cancer and prostatic intraepithelial neoplasia lesions [1,44]. Additionally, SENP1 expression directly correlated with prostate cancer aggressiveness and recurrence [11]. Furthermore, SENP1 overexpression was sufficient to induce cancer transformation of the normal prostate gland in mice, confirming its role in prostate carcinogenesis [1]. Mechanistically, SENP1 overexpression was associated with the increased expression of several transcription factors essential for the development and progression of prostate cancer, such as androgen receptor (AR) and HIF-1α, which is critical for neoangiogenesis [1,45,46]. Similarly, SENP2 aberrant activity influenced several transcription factors participating in cancer development and progression. SENP2 regulated the tumor suppressor p53 via deSUMOylation of its primary negative regulator, MDM2 [47,48]. Wnt signaling is one of the most prominent pathways for the regulation of cell proliferation, differentiation, and migration during development and homeostasis, as well as during tumorigenesis. SENP2 regulated β-catenin, which plays an essential role in the transduction of Wnt signaling in hepatocellular carcinoma and bladder cancer cells [18,19]. SENP2 inhibited the Notch and NF-κB signaling pathways in chronic lymphocytic leukemia cells, resulting in cell apoptosis [49]. In breast cancer cells, SENP2 participated in the regulation of estrogen receptor α (ERα) signaling and transforming growth factor (TGF-β) signaling, which modulated cancer cell proliferation, migration, and invasion [50,51].

The studies mentioned above indicate that SENPs are potential targets for anti-cancer treatment, especially against prostate cancer. The SENP-oriented inhibitors have been exclusively designed to target SENP1 and SENP2, the most clinically studied SENP members (Table 2). The discovery of SENP inhibitors is currently focused on the development of isoform-selective inhibitors, although this remains a highly challenging task. The similar amino acid sequence within the catalytic site, protein structure, and isopeptidase cleavage chemistry make it challenging to design isoform-selective SENP inhibitors. Contrary to SENP pan-inhibitors, isoform-selective inhibitors could display lower effective doses and higher drug safety, leading to improved therapeutic outcomes.

### 5.1. Natural and Non-Natural Peptide-Based Inhibitors

Initial strategies for designing SENP inhibitors were based on the use of natural and non-natural peptide backbones. Some of these inhibitors included SUMO-1 with an attached electrophilic trap (vinyl sulfone) [52], non-natural aza-peptide epoxides [53,54], and heptapeptide (FQQQTGG) equipped with a C-terminus Gly-derived fluoromethylketone moiety reassembling the Gly-Gly motif present in SUMO [55]. The aza-peptide epoxides included JCP-666, which was found by library screening of irreversible cysteine protease inhibitors [53,54]. JCP-666 harbors a reactive aza-epoxide that is susceptible to ring-opening in aqueous media. Therefore, a more stable analogue (VEA-260) was developed by removing the aspartic acid side-chain from the aza-epoxide scaffold. Even more potent SENP inhibitors based on the structure of JCP-666 and VEA-260 were synthesized with C-terminus acyloxymethyl ketone (AOMK) with a large *O*-acyl-anthracene group—the former as a mimetic of the peptide vinyl sulfone inhibitors [53]. Although the AOMK-based inhibitors, such as VEA499 and VEA561, generally significantly inhibited SENP1, 2, 6, and 7, they showed overall low cell permeability.

### 5.2. Non-Peptidyl Small Molecular Weight SENP Inhibitors

Due to peptide-based inhibitors’ poor pharmacokinetic properties, non-peptidyl small molecular weight SENP inhibitors have been developed. Non-peptidyl scaffolds generally improve stability and bioavailability, and thus benzodiazepine peptidomimetic inhibitors have been a reasonable synthetic direction [56]. Among benzodiazepines, compounds **36** and **38** were the most potent against SENP1 (IC_50_ of 15.5 and 9.2 μM, respectively). Additionally, these inhibitors suppressed prostate cancer (PC-3) cell growth in vitro with IC_50_ values of 13.0 and 35.7 μM, respectively. Another class of SENP inhibitors consists of 1-[4-(*N*-benzylamino)phenyl]-3-phenylurea derivatives based on a potent HIF-1α inhibitor [59]. The most potent compound **4** (GN6958) selectively inhibited SENP1 without affecting the closely related SENP2 with an IC_50_ of 29.6 μM. Inhibition of SENP1 proved effective in HeLa cells and resulted in the suppression of HIF-1α expression.

### 5.3. The Use of Virtual Screening for the Identification of SENPs Inhibitors

Several groups focused on the design and development of SENP inhibitors with a virtual screening-aided approach. These include 2-(4-chlorophenyl)-2-oxoethyl 4-benzamidobenzoate derivatives [57], 1,2,5-oxadiazoles [61], non-covalently binding sulfonyl-benzene non-natural amino acids [60], cell-permeable SENP specific inhibitor [62], and ebselen and 6-thioguanine [65]. Chen et al. reported the use of virtual screening for the identification of SENP inhibitors for the first time. They applied a SENP1 crystal structure from SENP1-SUMO-2-RanGAP1 complex and screened the SPECS library encompassing 180,000 compounds. The top 38 compounds were evaluated for biological activity using a SUMO-RanGAP cleavage assay combined with SDS-PAGE. The structure optimization of the top-ranked **J5** (IC_50_ of 2.39 μM) compound resulted in the synthesis of 2-(4-chlorophenyl)-2-oxoethyl 4-benzamidobenzoate analogues among which compounds **8d** and **8e** displayed similar effectiveness (IC_50_ of 1.175 μM and 1.080 μM, respectively) to the parent **J5** compound. At the same time, Madu et al. searched the 250,000 compound library provided by the Developmental Therapeutics Program (DTP) of the National Cancer Institute [60]. The inhibitory effect of 40 compounds was evaluated on SENP1 and SENP2’s ability to mature SUMO-1 and SUMO-2 precursors, and the most potent compounds contained sulfonyl-benzene groups. The inhibition of SENP1, 2, and 7 endopeptidase activity was evaluated using a bioluminescent reporter. The representative sulfonyl-benzene non-natural amino acid, SPI-01, displayed effective inhibition of isopeptidase activity in HeLa cells resulting in the accumulation of SUMO-2/3 conjugates as determined by SDS-PAGE. Importantly, combined NMR chemical shift perturbation analysis and enzyme kinetic experiments revealed that the inhibitory mechanism is mainly non-competitive and suggested that the inhibitor bound to the enzyme and the enzyme–substrate complex. SPI-01 was bound to the surface adjacent to the catalytic center that contacts the C-terminal end of the SUMO precursors. Kumar et al. searched a Namiki-shoji library of 4 million small molecule compounds to identify SENP2 inhibitors [61]. Eventually, 49 top hits were chosen for biological evaluation with FRET-based assay. Among these, compounds belonging to two scaffolds containing a 1,2,5-oxadiazole core were revealed, namely 2-phenoxy-*N*-(4-phenyl-1,2,5-oxadiazol-3-yl)acetamide and 2-phenoxy-*N*-[4-(2-phenoxyacetylamino)-(1,2,5-oxadiazol-3-yl)]acetamide. The most potent compound of each scaffold was compound **117** (IC_50_ of 3.7 µM against SENP2 vs. > 30 µM against SENP1) and compound **69** (IC_50_ of 5.9 µM against SENP2 vs. 9.7 µM against SENP1), respectively. Most compounds inhibited SENP1 and SENP2 with matching potencies and were specific to SENP, since no inhibition of other proteases, such as papain and trypsin, was observed. The SPECS database containing 200,000 small molecules was virtually screened by Wen et al. [62]. SENP1 structure docking residues 94–98 of SUMO-1 (i.e., QTGGH) were used for screening. Among the top 500 compounds, **117** were selected for the bioassay. SUMO-CHOP reporter assay revealed SI2 (IC_50_ of 1.29 µM) as the most potent inhibitor for SENP1. This was confirmed with an in vitro gel-based ∆RanGAP1-SUMO-2 cleavage assay. Cell-permeable pSI2 effectively inhibited the isopeptidase activity of SENP1, 2, and 3, but not SENP5, resulting in the accumulation of SUMOylated proteins in prostate cancer cells PC-3. Furthermore, SI2 was a specific SENP inhibitor since it had little effect on the activity of other cysteine proteases, such as cathepsin B and cathepsin D, and on proteasome activity, including chymotrypsin-like, trypsin-like, and caspase-like activity of the proteasome. Employing molecular docking, Wen et al., proposed a mechanism of inhibition where SI2 occupied a tunnel within SENP1, which guides the C-terminal tail of SUMO-1 toward the catalytic residues, thus preventing the binding of SUMO-1 to SENP1. Particularly, the chlorobenzene moiety of SI2 occupied the space where the Gly-Gly motif at the C terminus of SUMO-1 localized within SENP1. Similarly to Chen et al., Zhao et al., used the SPECS library and the Glide program to screen for SENP1 inhibitors [64]. Contrary to their predecessors, Zhao et al. used the co-crystal structure of SENP1 and pre-SUMO for calculations. The top 300 compounds from virtual screening were manually inspected to select 30 compounds for the SENP1 inhibitory assay. The RanGAP-SUMO cleavage assay revealed 11 compounds which functioned as SENP1 inhibitors. These inhibitors represent a diversity of scaffolds, providing a number of lead compounds. The structure optimization led to the development of **13m** (IC_50_ of 3.5 µM), with methoxybiphenyl terminus as the most potent inhibitor in the series.

### 5.4. Plant-Derived Inhibitors

An alternative to the virtual screening strategy for SENP inhibitor discovery is the study of anti-cancer compounds extracted from natural herbs. Many such compounds demonstrate pleiotropic anti-cancer effects. Triptolide is an active component extracted from the Chinese herb *Tripterygium wilfordii* Hook F with potent anti-cancer properties against some cancer types [58]. Triptolide inhibited SENP1 expression, resulting in enhanced SUMOylation in PC-3 cells and suppressing AR and c-Jun transcription. Furthermore, triptolide effectively induced cell death and cell cycle arrest in prostate cancer cells (LNCaP and PC-3) and suppressed xenografted PC-3 tumor growth in nude mice. Another natural SENP1 inhibitor is Momordin Ιc extracted from the dried fruit of *Kochia scoparia* (L.) [63]. Momordin Ιc inhibited SENP1 in PC-3 cells, resulting in the induction of cell cycle arrest. The study in a xenograft PC-3 tumor mouse model demonstrated that Momordin Ιc could suppress cell proliferation and induced cell death in vivo.

### 5.5. The Quantitative High-Throughput Cell-Free Screen to Identify SENP Inhibitors (AlphaScreen)

Bernstock et al. designed a quantitative high-throughput cell-free screen named AlphaScreen to identify SENP inhibitors [65]. A recombinant substrate for SENP2 was developed—SS3HS2 containing His-tag SUMO-2 conjugated to Strep-tag SUMO3DGG resembling a SUMOylated protein. Initially, more than 4000 compounds from both Sigma LOPAC and the NCGC Pharmaceutical Collection compound library were evaluated under AlphaScreen. The inhibitory efficacy of the selected 71 compounds was confirmed with cell-free SENP2 isopeptidase activity assay combined with SDS-PAGE. Among the 19 hits with complete inhibition of the catalytic domain, 13 were nontoxic compounds, as evaluated in rat neuroblastoma cell line B35. Since the study was designed to find nontoxic SENP2 inhibitors that would function as neuroprotective treatments for acute stroke, nontoxic compounds were screened to inhibit SENP2 and increase SUMO conjugation within the cell. Molecular docking calculations revealed that the top eight hits occlude the tunnel entrance, which guides the C-terminal tail of SUMO-1 toward the catalytic residues of the SENP2 structure, suggesting that the tested compounds impede the approach of the SUMO to the catalytic site. Among the most effective compounds, ebselen was tested in mice and increased global SUMOylation within the brain in vivo. Both the most potent compounds, ebselen and 6-thioguanine, were active against SENP1.

## 6. Conclusions and Perspectives

Given that post-translational modification of proteins, including SUMOylation, is a molecular regulatory mechanism involved in DNA damage repair, cell cycle progression, and carcinogenesis, it is not surprising that increasing attention has been given to SUMO-related pathways as potential anti-cancer targets. Inhibition of SUMO deconjugating enzymes—SENPs—is considered beneficial as a therapeutic approach in anti-cancer therapy. Until now, some SENP inhibitors have been analyzed utilizing different design and development strategies, including synthetic peptide-based compounds, virtual screening-assisted small molecular weight inhibitors, and natural compounds extracted from plants. Generally, SENP inhibitors’ development is in its infancy, and only a few compounds have been studied in cancer cells [56,58,59,62,63]. Some SENP inhibitors were developed as probe molecules to facilitate the study of the biological mechanism of SENPs exclusively [66]. Among cell-active compounds, two derived from natural products, triptolide and Momordin Ic, suppressed the proliferation of LNCaP and PC-3 prostate cancer cells and inhibited the xenografted PC-3 tumor growth in nude mice [58,63]. No other SENP inhibitor has been studied for in vivo efficacy against cancer, and none of them has reached clinical trials. Although the number of studies in cells is scarce, they implicate that the design of SENP inhibitors with therapeutic potential is achievable. However, most of the presently identified inhibitors’ therapeutic potential is limited due to their covalent binding to the active site cysteine and low specificity. In drug development strategies, there is a tendency to avoid covalent inhibitors to minimize the risk of unpredictable side effects, such as these provided by haptens and non-specific irreversible modification of off-target proteins. Most SENP inhibitors were developed to bind active site cysteine covalently. Due to a lack of structural data about non-enzymatic sites that can be targeted for SENP inhibition, the design and development of non-covalent small molecule inhibitors are hampered. Despite this inconvenience, virtual screening was successfully applied to find a new class of SENP non-competitive inhibitors [60]. Crystallographic studies revealed that a tunnel-like cavity guides the C-terminal tail of SUMO-1 toward SENPs’ active site. This allows for the accommodation of only a SUMO C-terminal Gly-Gly motif in the substrate cleft [67]. Electrophiles can irreversibly bind the active site cysteine, embedded in a typical catalytic triad (Cys-His-Asp), in all SENPs; however, this approach does not lead to the development of selective inhibitors. The common characteristic of the SENP family is their conserved catalytic domain of approximately 200 amino acids at the C-terminus. SENP family members share 20 to 60% sequence identity within their catalytic domains [68]. The SENP1–SENP2, SENP3–SENP5, and SENP6–SENP7 pairs show the highest degree of similarity to each other. The structural resemblance within families of cysteine proteases impedes a design of inhibitors with sufficient selectivity. Only two SENP inhibitors (SI2 and compound **4**) proved to act selectively on the target [59,62]. Among the SENP isoforms, the catalytic domains of SENP6 and SENP7 are the most diverse; however, their biological function and therapeutic relevance are yet to be described.

A computer-assisted approach, including virtual screening and molecular docking, identified some lead compounds in recent years. The availability of crystal structure and anticipated therapeutic potential channeled SENP inhibitor development mostly against SENP1 and SENP2. The availability of sufficient structural information about SENPs, including crystal structures in an apo form (not ligand-bound) and in complexes with different SUMO precursors and isopeptide-linked SUMO-RanGAP1 conjugates, should facilitate computational methods for the identification of non-covalent selective inhibitors. However, many new chemical entities will have to be identified and optimized since SENP inhibitors are beginning their road to successful implementation into clinical practice.

## Author Contributions

P.T. wrote the manuscript and created the figure and tables. K.W. critically reviewed the manuscript. Both authors have read and agreed to the published version of the manuscript.

## Figures and Tables

**Table 1:** SENP proteases in clinical and molecular studies.

SENP Protease	Main Localization	Enzymatic Activity	Cancer/RNA Expression *	Clinical Studies	Molecular Studies	References
SENP1	Nucleoplasm	C-terminal hydrolase, isopeptidase	Breast	polymorphism c.1691 + 36C > T (rs12297820) was associated with risk of metastases		[16]
			Colorectal/↑	not related to tumor invasion, lymph node involvement or tumor cell differentiation	regulates cell cycle via CDK inhibitors (p16, p19, p21, and p27)	[13]
			Myeloma/↑		regulates sensitivity to apoptosis, proliferation, and colony formation regulates NF-κB signaling	[17]
			Liver/↑	prognostic marker		TCGA
			Neuroblastoma/↑	overexpressed in metastatic tissues vs. primary tumor tissue	promotes cell invasion and migration regulates the expression of CDH1, MMP-9, and MMP-2	[12]
			Pancreatic/↑	correlates with lymph node metastasis and TNM stage	up-regulates MMP9	[8]
			Prostate/↑	correlates with cancer aggressiveness and recurrence	androgen receptor and hypoxia-induced stabilization of HIF1α and overexpression of downstream proteins (MMP2/MMP9)	[1,11]
			Renal/↑	prognostic marker		TCGA
SENP2	Nuclear pore complex	C-terminal hydrolase, isopeptidase	Bladder/↓		decreases cell migration and invasion inhibits the expression of MMP-13	[18]
			Breast	polymorphism c.902C > A, p.Thr301Lys (rs6762208) was associated with cancer occurrence		[16]
			Endometrial/↑	prognostic marker		TCGA
			Liver/↓		suppresses growth and colony formation modulates the stability of β-catenin	[19]
SENP3	Nucleolus	Isopeptidase	Gastric/↑		promotes epithelial-mesenchymal transition, cell migration, and metastasis potentiates the transcriptional activity of FOXC2	[9]
			Head and neck/↑	correlates with tumor differentiation	correlates with ROS	[20]
			Pancreatic/↑	prognostic marker		TCGA
SENP5	Nucleolus	C-terminal hydrolase, isopeptidase	Bone/↑		promotes cell growth, its inhibition results in cell cycle arrest and apoptosis via the regulation of cyclin B1 and caspase 3/7	[10]
			Breast/↑	negatively correlates with survival	associates with cell proliferation, migration, invasion, and colony formation regulation of phenotype through SENP5-TGFb-MMP9 cascade	[6]
			Endometrial/↑	prognostic marker		TCGA
			Head and neck/↑	associates with tumor differentiation	protects cells from oxidative stress-induced apoptosis through the stabilisation of mitochondria	[21,22]
			Liver/↑	prognostic marker		TCGA
			Renal/↑	prognostic marker		TCGA
SENP6	Nucleoplasm	Isopeptidase, chain editing	Liver/↑		silencing SENP6 causes sensitisation to radiation and inhibition of cell proliferation required for radiation-induced NF-κB activation	[15]
			Renal/↑	prognostic marker		TCGA
			Thyroid/↑	prognostic marker		TCGA
SENP7	Nucleoplasm	Isopeptidase, chain editing	Head and neck/↑	prognostic marker		TCGA

* RNA expression in cancer cells vs. adjacent normal tissue. TCGA—RNA-seq data from The Cancer Genome Atlas (TCGA) on 19th January 2021. ↑ RNA overexpression. ↓ Down-regulation of RNA expression.

**Table 2:** SENP Proteases Inhibitors.

Inhibitor Name	Target Protein	Compound Name/Source	IC_50_ (µM)	Biological Activity	References
SUMO-1-VS	SENP2	SUMO-1-vinyl sulfone		Interacted directly with SENP2 in its catalytic site as verified with SDS-PAGE.	[52]
JCP666	SENP1 SENP2	Electrophilic aza-peptide epoxide with non-natural peptide backbone	13.8 7	Virtual screening-aided design. Included aza-aspartic acid epoxide with the bulky di-naphthyl amide susceptible to ring opening in aqueous media. SENP inhibition evaluated with ProSUMO processing assay combined with SDS-PAGE and a cleavage assay with SUMO-conjugated fluorogenic substrate.	[53,54]
VEA260	SENP1 SENP2	JCP666 analogue without aspartic acid side-chain	7.1 3.7	SENP inhibition evaluated with ProSUMO processing assay combined with SDS-PAGE and a cleavage assay with SUMO-conjugated fluorogenic substrate.	[53,54]
VEA499 VEA561	SENP1 SENP2 SENP2 SENP6 SENP7	Acyloxymethyl ketone (AOMK)-based compounds which retained the overall structure of VEA260 and JCP666	3.6 0.25 5.7 4.2 4.3	AOMKs equipped with a large *O*-acyl-anthracene group—mimetics of the peptide vinyl sulfone inhibitors. VEA499 and VEA561 based on natural peptide sequences. VEA499 with sequence of SUMO-1 (QTGG) was most potent for hSENP1 and hSENP2, and VEA561 with the ubiquitin sequence (LRGG) was the most potent against hSENP6 and hSENP7. Enzymatic activity evaluated with a cleavage assay with SUMO-conjugated fluorogenic substrate. Low cell permeability.	[53]
*N*-acetylglycine fluoromethylketone (Compound 1)	SENP1 SENP2	Glycine fluoromethylketone (G-FMK) with peptide sequence	5–10 5–10	G-FMK equipped with peptide sequence (FQQQTGG) specific to SUMO-2/3. G-FMK acted as SENP-specific activity based probe. It shared binding site for SENP1 with SUMO-1. Direct interaction between G-FMK and SENP1/2 assayed with activity-based labeling combined with SDS-PAGE. G-FMK targeted SENP1 and SENP2 in HEK293A cell lysates.	[55]
Compound **36** Compound **38**	SENP1	Benzodiazepines	15.5 9.2	Compounds screened for SENP1 inhibition with SUMO-ΔRanGAP cleavage assay combined with SDS-PAGE. Compounds **36** and **38** inhibited the growth of prostate cancer cells (PC-3) with IC_50_ values of 13.0 and 35.7 μM, respectively.	[56]
**J5** Compound **8d** Compound **8e**	SENP1	2-(4-Chlorophenyl)-2-oxoethyl 4-benzamidobenzoate derivatives	2.385 1.175 1.080	Developed with virtual screening. Molecular docking showed that **J5** fitted in the SENP1 binding site. The SENP1 inhibitory potency was evaluated with SUMO-ΔRanGAP cleavage assay combined with SDS-PAGE.	[57]
Triptolide	SENP1	Diterpene lactone extracted from the Chinese herb *Tripterygium wilfordii* Hook F	0.0203 (PC-3) 0.009754 (LNCaP)	Inhibited proliferation and induced cell death in prostate cancer cells (LNCaP and PC-3). Suppressed xenografted PC-3 tumor growth in nude mice. Down-regulated SENP1 and c-Jun expression in PCa cells and androgen receptor expression in LNCaP cells. Down-regulation or over-expression of SENP1 inhibited triptolide anti-cancer efficacy.	[58]
Compound **4** (GN6958)	SENP1	1-[4-(*N*-benzylamino)phenyl]-3-phenylurea derivative	29.6	Directly interacted with SENP1 in cells as evaluated with the use of HP SpinTrap affinity column combined with SDS-PAGE. Inhibited SENP1 enzymatic activity as assayed with fluorogenic substrate SUMO-1-AMC. Specific inhibitor, did not inhibit SENP2. Suppressed HIF-1α accumulation in HeLa cells.	[59]
SPI-01	SENP1 SENP2 SENP7	sulfonyl-benzene non-natural amino acid	5.9 2.9 3.5	Virtual screening was used for the study. SPI-01 inhibited the isopeptidase activities in cells as demonstrated with DUB-Glo assay. Inhibitory mechanism is mainly non-competitive as demonstrated with DUB-Glo enzyme kinetic experiments and NMR binding analysis.	[60]
Compound **117** Compound **69**	SENP2 SENP1 SENP2 SENP1	1,2,5-oxadiazoles	3.7 >30 5.9 9.7	Compound development with virtual screening. FRET-based assay for quantification of endopeptidase activity.	[61]
SI2	SENP1 SENP2 SENP3	Biphenyl-4-carboxylic acid ester with chlorobenzene moiety	1.29	Compound selection with hierarchical virtual screen. Cell-permeable SENP specific inhibitor. Occupied a tunnel in the catalytic centre of SENP1.	[62]
Momordin Ic	SENP1	Natural pentacyclic triterpenoid extracted from various sources such as *Kochia scoparia* (L.)	15.37	Inhibited SENP1 in cells as shown with SUMO-2-ΔRanGAP1 cleavage assay combined with SDS-PAGE. Direct interaction with SENP1 in cells determined with cellular thermal shift assay. Inhibited prostate cancer PC-3 cell proliferation. Suppressed cell proliferation and induced cell death in a xenograft PC-3 tumor mouse model.	[63]
Compound **13m**	SENP1	4′-methoxy-biphenyl-3-carboxylic acid 3-(3-phenylpropionylamino)-benzylamide	3.5	Designed with virtual screening. SENP1 inhibition determined by SUMO-RanGAP cleavage assay combined with SDS-PAGE.	[64]
Ebselen and 6-thioguanine	SENP2	synthetic organo-selenium compound		Virtual-screening-assisted strategy of drug identification. Molecular docking calculations demonstrated that ebselen occluded the entrance to the SENP2 tunnel. Both ebselen and 6-thioguanine were non-cytotoxic, increased SUMO conjugation in B35 neuroblastoma cells, and protected the cells from OGD (in vitro stroke model). Ebselen upregulated global SUMOylation within the brains of mice. Both compounds inhibited SENP1.	[65]
